# Data on solar sunburning ultraviolet (UVB) radiation at an urban Mediterranean climate

**DOI:** 10.1016/j.dib.2017.02.053

**Published:** 2017-03-11

**Authors:** Katerina G. Pantavou, Constantinos P. Jacovides, Georgios K. Nikolopoulos

**Affiliations:** a Department of Environmental Physics and Meteorology, Faculty of Physics, National & Kapodistrian University of Athens, University Campus, Zografou, Building Physics 5, 157 84 Athens, Greece; b Medical School, University of Cyprus, Nicosia, Cyprus

**Keywords:** Ultraviolet, UVB, Radiation, Sunburn, Field survey

## Abstract

This article describes data on the intensity of ultraviolet B (UVB) radiation collected during field questionnaire-based surveys in Athens, Greece. The surveys were conducted over 11 days of July and October 2010 at three different urban, outdoor sites. A total of 1104 interviews were conducted. The participants were asked to report whether they felt they got a sunburn at the moment of the interview. Questions related to personal characteristics including skin type and exposure time (visit duration at the interview site) were also included in the questionnaire.

**Specifications Table**

**Table:** 

Subject area	*Environmental Science/Biometeorology*
More specific subject area	*Solar irradiance*
Type of data	*Excel spreadsheet*
How data was acquired	*Data were collected during field questionnaire-based surveys. Measurements of the intensity of ultraviolet B radiation (UVB, also called SUV – Sunburning UV) in Minimal Erythemal Doses per Hour (MED/h) were taken using a UV MINDER® Model 3D UV intensity meter (Solar Light Co). Subjective responses were recorded through questionnaire-based interviews.*
Data format	*Raw*
Experimental factors	*The participants were people passing by or visiting the monitoring sites.*
Experimental features	*The field surveys were conducted at three different sites of the metropolitan area of Athens: Syntagma square, Ermou street and Flisvos coast, during summer and autumn 2010.*
Data source location	*Athens (37°59′20″N, 23*°*43′41″E), Greece*
Data accessibility	*Data are with this article.*

**Value of the data**

• SUV data and individuals’ responses of getting a sunburn can be used to examine the perception of individuals in terms of solar radiation and determine thresholds related to uncomfortable and potentially detrimental conditions.
• The comparison of this dataset with others in similar or different climates or even in different settings e.g. at a beach, could provide insights in understanding public perception of solar radiation and promoting solar radiation awareness.
• The data may allow the comparison with Global Solar UV Index (UVI) [1] contributing to appropriate individual behaviors and attitudes towards sun safety.
• The data could be used to examine the relationship between the intensity of ultraviolet B radiation and total ozone column.

## 1 Data

This article includes data on the intensity of UVB (SUV – Sunburning UV) in Minimal Erythemal Doses per Hour (MED/h), and on the subjective assessment of getting a sunburn as reported through questionnaires filled in by 1104 individuals along with some personal characteristics including clothing color, standing or not standing under the sun during the interview, skin type, and part of the body sunburned. The dataset is in an Excel file, SUVdata.xlsx.

## 2 Experimental design, materials and methods

The field surveys were conducted over 6 days in July and 5 days in October 2010 at three outdoor urban sites of Athens: Syntagma square, Ermou street and Flisvos coast (Table 1). The participants were Caucasian in race.

Syntagma square is located in the center of Athens surrounded by multistore buildings. It contains green spaces and a fountain. Ermou Street is a shopping street in Athens, mostly used by pedestrians. Flisvos Coast is located in the southern suburbs of Athens and next to a densely populated urban area. Data were collected on two days for each site and season. On one day data were collected from morning to mid-day and on the other day from afternoon to evening and night hours, except for the Flisvos coast in autumn when surveys were carried out only in the afternoon. The intensity of UVB (SUV – Sunburning UV) in Minimal Erythemal Doses per Hour (MED/h) was measured at the height of 1.1 m above the ground (average height of the center of gravity of the human body) using a mobile tripod. People passing by or visiting the monitoring sites were interviewed based on a structured questionnaire (Table 2). The questionnaire included information on gender, age, color of participants’ clothes, duration of visit at the interview site, and on wearing or not sunglasses or a hat. The participants were also asked to report whether they felt they got a sunburn at the moment of the interview and to self-evaluate their skin type in accordance to the Fitzpatrick Skin Type classification [2]. The SUV measurement was recorded on the questionnaire at the time each interview started.

## Figures and Tables

**Table 1:** Time frame in local time (Greenwich Mean Time +03:00) and meteorological conditions during the days of the field surveys. Average daily air temperature (T_air_), relative humidity (RH) and wind speed (WS) were recorded at Thissio Station (Institute of Environment and Sustainable Development, National Observatory of Athens).

Season	Date	Start time	End time	Site	Average daily values		
					T_air_ (°C)	RH (%)	WS (m·s^−1^)
Summer	15/07/2010	16:45	19:30	Syntagma square	31.6	45	2.3
	16/07/2010	15:58	20:30	Ermou street	30.7	44	6.3
	17/07/2010	19:13	20:20	Flisvos coast	30.7	36	6.4
	18/07/2010	11:33	13:50	Flisvos coast	30.8	36	3.9
	20/07/2010	10:05	15:18	Syntagma square	31.0	40	3.6
	21/07/2010	10:40	14:06	Ermou street	30.0	47	2.6
Autumn	16/10/2010	10:21	15:05	Ermou street	25.9	72	1.7
	17/10/2010	11:03	15:09	Syntagma square	26.9	72	2.0
	20/10/2010	16:15	18:30	Syntagma square	24.8	64	4.0
	23/10/2010	16:23	18:32	Ermou street	18.7	65	1.7
	24/10/2010	13:54	15:57	Flisvos Coast	22.0	64	1.5

**Table 2:** The questionnaire used in the field surveys. Data in file SUVdata.xlsx. are coded according to the numbers denoted in parentheses.
